# Analysis of anatomical variations of intrapelvic vessels for advanced pelvic surgery

**DOI:** 10.1186/s12893-020-00711-0

**Published:** 2020-03-16

**Authors:** Atsushi Hamabe, Takashi Harino, Takayuki Ogino, Tsukasa Tanida, Shingo Noura, Shunji Morita, Keizo Dono

**Affiliations:** Department of Surgery, Toyonaka Municipal Hospital, 4-14-1 Shibahara-cho, Toyonaka, Osaka, 560-8565 Japan

**Keywords:** Pelvic anatomy, Iliac artery, Iliac vein, Rectal cancer

## Abstract

**Background:**

In pelvic surgery, it is important to anticipate potential anatomic variations, which may be unknown, and inter-relationships among intrapelvic vessels. Here, we comprehensively analyzed intrapelvic vessel patterns.

**Method:**

This retrospective analysis included 81 patients that underwent colorectal surgery in our institution in 2016. A total of 162 half-pelvises were imaged with contrast-enhanced computed tomography. We scrutinized thin-slice images.

**Results:**

We found variations in the number of internal iliac veins. In 47.5% of cases, one internal iliac vein drained into the ipsilateral common iliac vein in both halves of the pelvis. In the other cases, several internal iliac veins were observed in one or both halves of the pelvis. We analyzed the inter-relationships between the superior gluteal artery and the sacral nerve plexus in pelvic halves. Superior gluteal arteries ran between the 5th lumbar nerve and 1st sacral nerves, in 82% of halves, and lateral to the 5th lumbar nerve, in 17% of halves. Dorsally, the superior gluteal artery ran on the medial side of the internal iliac vein in 15% of halves. In 28% of half-pelvises, two superior gluteal veins were observed. Superior gluteal veins passed through the sacral nerve plexus lateral to 5th lumbar, between 5th lumbar and 1st sacral, and between 1st and 2nd sacral nerve, in 42.0, 47.5, and 37.7% of halves, respectively. We evaluated the rate of symmetric pelvic anatomies, and found that all anatomic variations formed symmetrically, except the number of internal iliac veins.

**Conclusion:**

This study clarified the anatomical variations of intrapelvic vessels and their inter-relationships. These findings will benefit our understanding of pelvic anatomy and enhance the safety of radical surgery for treating pelvic diseases.

## Background

Many organs and tissues lie in complicated juxtaposition in the pelvic cavity, including vessels, nerves, muscles, urogenital organs, and the rectum. The spatial configurations of these organs are difficult to comprehend for many surgeons. Recent studies have demonstrated that three-dimensional models or three-dimensional simulations could be helpful in understanding this complexity [1, 2]. In performing advanced surgery for rectal cancer and cancers that arise in urological or gynecological organs, it is necessary to have an accurate, comprehensive understanding of pelvic anatomy to perform safe, oncologically appropriate surgery. However, in addition to the complexity of the pelvic cavity, many anatomical variations, particularly vascular patterns, make it even more difficult to understand. Only a few studies have demonstrated the morphological variability in the courses of the internal iliac artery, vein, or obturator vessels. Moreover, most of those studies were based on a small series or they addressed the variability of an individual artery or vein [3–9]. During surgery, it is also quite important to understand the inter-relationships between intrapelvic organs, in addition to individual variability, and these issues are rarely studied. Based on this background, the current study was undertaken to provide a comprehensive analysis of intrapelvic vascular anatomic variability, to elucidate the distribution of different pelvic vascular pattern variations, and to determine systematically the inter-relationships among intrapelvic vessels.

## Methods

### Patients

This retrospective analysis included 81 patients that underwent colorectal surgery for colorectal cancers in our institution in 2016. These patients had undergone contrast-enhanced computed tomography (CT) in a preoperative work-up. All CT images were acquired with a 64-detector row CT scanner (Revolution GSI and Revolution EVO, GE Healthcare, Milwaukee, WI, USA). The CT scan was started at 70 s after an injection of non-ionic contrast agent with iodine. In our institution, we routinely reconstructed thin-slice CT images (1.25-mm thick or occasionally 0.625-mm thick) for rectal cancer cases that required lateral pelvic lymph node dissection.

### Interpretation of intrapelvic vascular anatomy

A total of 162 pelvic halves in 81 patients were examined. CT images were interpreted in detail, independently, by two surgeons (AH, a specialist in colorectal surgery, and TH). In cases of disagreement, the final interpretation was based on a mutual agreement between the two surgeons. After this process, all images were carefully confirmed again (by AH).

We examined the locations of spinal nerves, 5th lumbar nerve (L5), 1st sacral nerve (S1), and 2nd sacral nerve (S2), which join together to form the sacral nerve plexus. We also examined the locations of arteries, including the common, external, and internal iliac arteries (CIA, EIA, and IIA, respectively), the superior and inferior gluteal arteries (SGA and IGA, respectively), and the internal pudendal artery (IPA). We also examined the locations of veins, including the common, external, and internal iliac veins (CIV, EIV, and IIV, respectively), the superior gluteal vein (SGV), and the aberrant obturator vein.

**Fig. 1:**
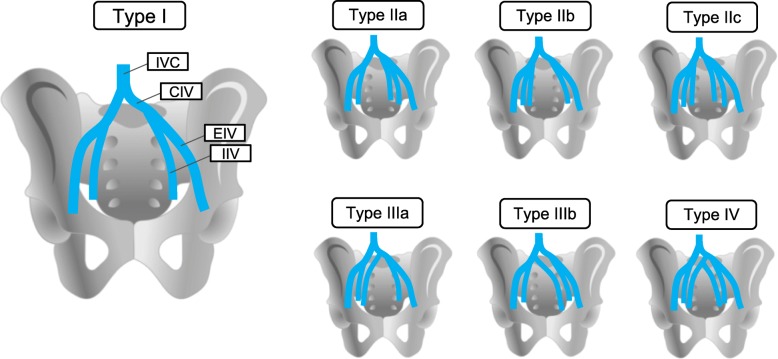
Variations in the branching pattern of the internal iliac vein. IVC, inferior vena cava; CIV, common iliac vein; IIV, internal iliac vein; EIV, external iliac vein

In the current study, variations in the branching pattern of the IIV were classified as follows (Fig. 1): in type I, one IIV drained into the ipsilateral CIV in both halves of the pelvis; in type II, two IIVs drained into the ipsilateral CIV in one or both halves of the pelvis; in type III, one of the two IIVs drained into the contralateral CIV, and the other drained into the ipsilateral CIV; and type IV comprised all variations in IIV patterns that did not fit into types I-III. Additionally, type II was subclassified into three subtypes: in type IIa, two IIVs were present in the left half of the pelvis; in type IIb, two IIVs were present in the right half of the pelvis; and in type IIc, two IIVs were present in both halves of the pelvis. Similarly, type III was classified into two subtypes: in type IIIa, one IIV draining toward the ipsilateral cavity ran from the right cavity into the left CIV; in type IIIb, it ran from the left cavity into the right CIV.

### Statistical analysis

All statistical analyses were performed with JMP pro 13.0.0 software (SAS Institute, Cary, NC, USA). A kappa score was calculated to evaluate whether the pelvic anatomy was symmetric.

## Results

### Patient background

The cohort of 81 patients included 42 (51.9%) males and 39 (48.1%) females, with a median age of 73 years (range 40 to 84 years). The primary tumors were located in the right-side colon (from the cecum to the transverse colon) in 19 patients (23.5%); in the left-side colon (from the descending to the sigmoid colon) in 30 patients (37.0%); and in the rectum in 32 patients (39.5%). CT slices were 0.675-mm thick in 7 cases (8.6%) and 1.25-mm thick in 74 cases (91.4%).

### Internal iliac vein

The vascular branching pattern variations observed in the IIV are shown in Fig. 1 (details in Table 1). The type I branching pattern was observed in 47.5% of all cases. The other cases had several IIVs in one or both halves. Types II and III branching patterns were observed in about 40 and 10% of all cases, respectively. Among 10 cases of type III, 9 were type IIIa. One case of type IV was observed, where two IIVs were present on both sides, and two medial IIVs joined together in front of the sacrum, then drained into the left CIV.

**Table 1:** Vascular pattern variability in the pelvis

Analyzed variations	**Types**	**Number of patients**	**%**
Branching pattern of internal iliac vein^a^	I	39	48.1%
	IIa	11	13.6%
	IIb	13	16.0%
	IIc	7	8.6%
	IIIa	9	11.1%
	IIIb	1	1.2%
	IV	1	1.2%
Analyzed variations	**Types**	**Number of pelvic halves**^**c**^	**%**
Section of the route in sacral nerve plexus, through which the SGA passes^b^	Lateral to L5	27	16.7%
	L5-S1	133	82.1%
	S1-S2	2	1.2%
Section of the route in sacral nerve plexus, through which the SGV passes^b^	Lateral to L5	68	42.0%
	L5-S1	77	47.5%
	S1-S2	61	37.7%
	Medial to S2	2	1.2%
Inter-relationship between the IGA and the IPA at the exit of the pelvis	IGA medial and IPA lateral	104	64.2%
	IGA lateral and IPA medial	13	8.0%
	IGA and IPA as a common trunk	19	11.7%
	Others	26	16.0%
Aberrant obturator vein	Present	114	70.4%
	Absent	48	29.6%

^a^These patterns are illustrated in Fig. 1. ^b^These patterns are illustrated in Fig. 2. ^c^In some cases, two SGVs were found in a single pelvic half; therefore, the total number in that section exceeds the 162 pelvic halves, and the percentage exceeds 100%. *IGA* Inferior gluteal artery, *IPA* Internal pudendal artery, *L5* 5th lumbar nerve, *S1* 1st sacral nerve, *S2* 2nd sacral nerve, *SGA* Superior gluteal artery; SGV, superior gluteal vein

### Inter-relationships between the sacral nerve plexus and superior gluteal vessels

**Fig. 2:**
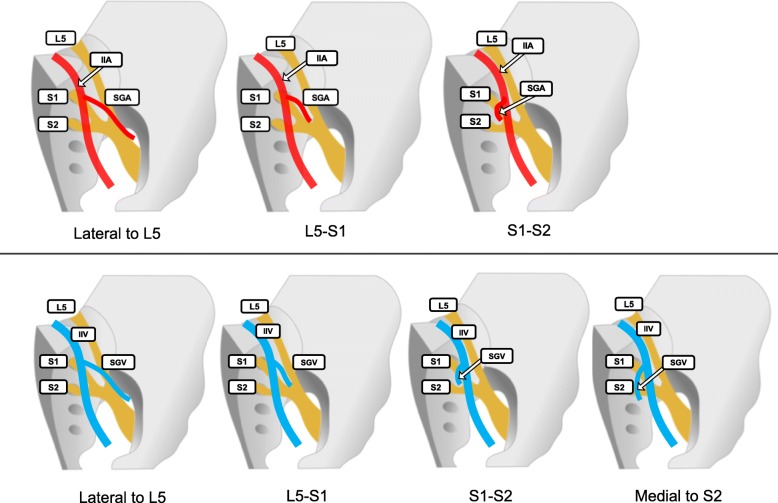
Variations in inter-relationships between the spinal nerves and the superior gluteal artery and vein. (Upper panel) Patterns of the superior gluteal artery (SGA). (Lower panel) Patterns of the superior gluteal vein (SGV). IIA, internal iliac artery IIV, internal iliac vein; L5, 5th lumbar nerve; S1, 1st sacral nerve; S2, 2nd sacral nerve

On each side of the pelvis, there are four routes for vessels to exit out of pelvis formed by the L5, S1, and S2 spinal nerve branches. The vascular branches of the internal iliac vessels must negotiate these routes as they pass out of the pelvis. We examined the inter-relationships between these routes and the SGA. We found that 82% of SGAs ran between the L5 and S1 branches, and 17% of SGAs ran lateral to the L5 branch (Fig. 2, upper panel; Table 1). Similarly, we analyzed the inter-relationships between the routes and the SGV. In 28% of pelvic halves, two SGVs were observed; therefore, the route through which each SGV passed was evaluated independently (Fig. 2, lower panel; Table 1). We found that the SGVs passed through the routes lateral to L5, between the L5 and S1, and between the S1 and S2 in 42.0, 47.5, and 37.7% of the halves, respectively.

### Inter-relationships among superior gluteal artery, internal iliac artery, and internal iliac vein

**Fig. 3:**
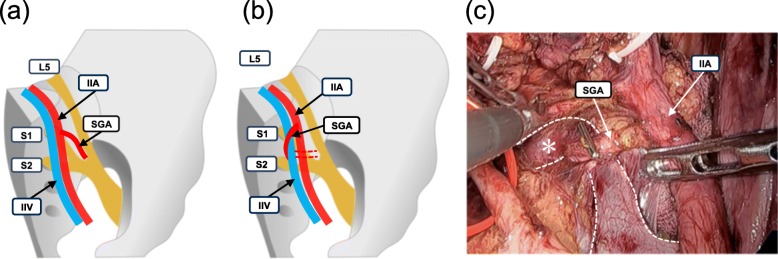
Inter-relationships among the superior gluteal artery (SGA) and the internal iliac artery and vein. (a) The SGA (red) runs dorsal, on the lateral side of the internal iliac vein (blue). (b) The SGA runs dorsal, on the medial side of the internal iliac vein. (c) Representative photograph of the right side of the pelvic cavity, which shows the pattern described in (b). Photograph was acquired during laparoscopic surgery for a lateral lymph node dissection. Asterisk (*) represents the internal iliac vein. IIA, internal iliac artery IIV, internal iliac vein; L5, 5th lumbar nerve; S1, 1st sacral nerve; S2, 2nd sacral nerve; SGA, superior gluteal artery

We investigated the inter-relationships among the SGA, IIA, and IIV (Fig. 3). The SGA ran dorsally, on the lateral side of the IIV in 85% of all pelvic halves. In the remaining 15% of pelvic halves, the SGA ran dorsally, on the medial side of the IIV (Fig. 3a, b and c).

### Unique variation

**Fig. 4:**
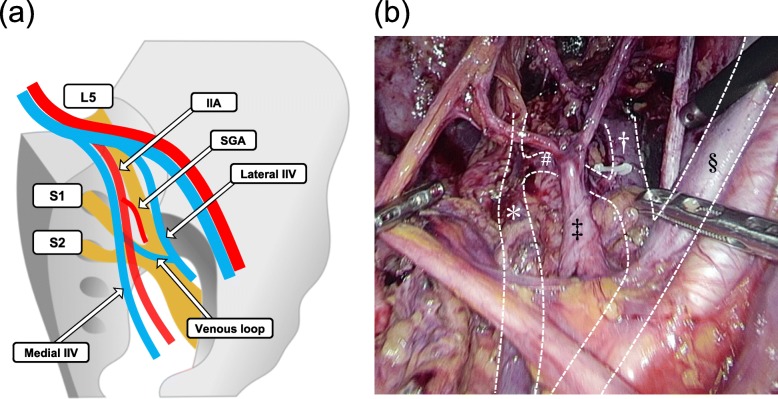
A unique variation of the inter-relationship between the internal iliac artery (IIA) and vein (IIV). (a) The schematic figure illustrates this pattern. (b) Representative intra-operative photograph shows this pattern on the right side of the pelvic cavity. Photograph was acquired during laparoscopic surgery for a lateral lymph node dissection. Asterisk (*) represents the medial internal iliac vein (IIV); dagger (^†^) represents the lateral IIV; double dagger (^‡^) represents the internal iliac artery (IIA); hash sign (^#^) represents the venous loop between the two IIVs, which has formed in front of the sacral nerve plexus; and the section sign (^§^) represents the external iliac vein. IIA, internal iliac artery IIV, internal iliac vein; L5, 5th lumbar nerve; S1, 1st sacral nerve; S2, 2nd sacral nerve; SGA, superior gluteal artery

Fifteen pelvic halves showed a unique pattern in the inter-relationship between IIA and IIV (Fig. 4a and b). In these halves, two IIVs intercommunicated with each other, and thus, they formed a venous loop in front of the sacral nerve plexus. The SGA passed through this loop, and then ran dorsally, toward the buttock.

### Vascular variations in lateral cavities

Furthermore, we investigated additional variations, including the inter-relationship between the IGA and IPA, at the level of the pelvic outlet, and the presence of an aberrant obturator vein (Table 1). About 70% of the IIAs divided into IPA and IGA branches in the pelvic cavity. In 12% of the pelvic halves, the IIAs passed out of pelvis as a common trunk. In pelvic halves where IPA and IGA were divided in pelvic cavity, the IGA exited the pelvis on the medial side more frequently than on the lateral side of the IPA. The remaining cases had other types of IIA divisions; for example, sometimes, the IGA branched off the SGA outside the pelvis and ran toward the buttock. An aberrant obturator vein was present in 70% of pelvic halves.

### Anatomic symmetry in the lateral pelvic cavities

The rate of pelvic anatomic symmetry, or the concordance rate of pelvic anatomies, was analyzed for the following variations; the route that the SGA passed through; a SGA that ran dorsally, on the medial side of the IIV; the number of SGVs; the route that the SGV passed through; the presence of an aberrant obturator vein; and the number of IIVs (Table 2). We found that all anatomical variations, other than the number of IIVs, were symmetric.

**Table 2:** Anatomic symmetry in the lateral cavities of the pelvis

Analyzed variations	Concordance rate	Kappa	***P*** value
The route where the SGA passes through	77.8%	0.26	0.0069
SGA runs dorsally, on the medial side of the IIV	84.0%	0.39	< 0.0001
The number of SGVs	70.4%	0.28	0.0057
The SGV passes lateral to L5	75.3%	0.49	< 0.0001
The SGV passes between L5 and S1	71.6%	0.43	< 0.0001
The SGV passes between S1 and S2	74.1%	0.45	< 0.0001
The SGV passes medial to S2	100.0%	1	< 0.0001
The presence of an aberrant obturator vein	76.5%	0.45	< 0.0001
The number of IIVs	61.7%	0.13	0.1061

*IIV* Internal iliac vein, *L5* 5th lumbar nerve, *S1* 1st sacral nerve, *S2* 2nd sacral nerve, *SGA* Superior gluteal artery, *SGV* Superior gluteal vein

## Discussion

This study revealed details about the inter-relationships among pelvic vessels and their anatomic variations in pelvic cavity. Furthermore, several unique anatomical variations were observed in a few cases. This study was the first to elucidate these patterns, and our findings have important implications for surgeons.

Since Adachi first classified the IIA branching variations into five types [3], several studies have investigated the distributions of branching patterns in different populations [4–6]. Although those studies analyzed arterial variations in detail, they only assessed the IIA, independent of other vessels. Information other than arterial variations have not been available, due to the scarcity of studies that investigated vein, muscle, or nerve anatomies. Therefore, the present study addressed the unmet need for a comprehensive analysis of pelvic vascular anatomy, including anatomic inter-relationships and variations.

Current textbooks on human anatomy illustrate intrapelvic vessels and their inter-relationships as invariant [10, 11]. Typically, the SGA branches off the IIA, runs posteriorly, on the lateral side of the IIV, passes between the L5 and S1 nerves, and runs toward the buttock. In addition, the IIV branches off the CIV, one on each side. Many surgeons probably consider this configuration to be typical pelvic anatomy, and they might implement surgery based on that assumption. However, the present study demonstrated that not all pelvic vessels form those stereotypic patterns. In locally recurrent rectal cancer treatments, surgical resection is one of the options for cure, in which we must perform radical surgery with en bloc resection with involved surrounding organs including internal iliac vessels to achieve tumor-free resection [12–14]. Understanding the above vascular variations might be valuable to prevent inadvertent intraoperative bleeding from the internal iliac vessels in such advanced surgeries.

In our opinion, some of the facts clarified in this study are quite noteworthy. First, we described the anatomic inter-relationships around the IIA. Although the SGA branches off the IIA and typically passes through the L5-S1 split, 17% of SGAs run in front of L5 and pass lateral to L5. In addition, in some cases, the SGA runs dorsally, on the medial side of the IIV. These uncommon variations should be recognized preoperatively to prevent unexpected vascular injury, which might lead to severe outcomes. For example, lateral lymph node dissection is needed to resect advanced rectal cancer with metastatic lateral node [15, 16]. During this procedure, lymph nodes around IIVs are included in the dissected area, and therefore we must pay attention to the SGA running on the medial side of the IIV which was found to be observed in 15% of pelvic halves. Second, we found that pelvic anatomy was formed symmetrically. This finding suggested that, although many anatomic variations are present in the pelvis, pelvic anatomies are not likely to arise at random. These facts might facilitate the evaluation and detection of anatomic variations in the pelvis with more precision, in advance of surgery.

Although most preceding studies were based on cadaver inspections, in this study, we assessed contrast-enhanced CT images. In cadaver inspection, after dissecting the anatomic structures, it is not necessarily simple to determine the original structure. The procedure might also obscure anatomic inter-relationships. Furthermore, recent advances in CT technology have allowed clear depictions of anatomic structures even when they were covered with visceral fat. Several studies that analyzed abdominal vascular anatomy also adopted CT for making assessments [17, 18].

This study had several limitations. First, the retrospective study design had inherent limitations. In particular, our findings could not be confirmed intra-operatively. Second, the CT scans were performed once in the portal phase; this technique could have obscured the depiction of some individual vessels. Ideally, both arterial and venous phases should be obtained.

## Conclusions

This study clarified the anatomic variations and inter-relationships of pelvic vessels in detail. We found some rare patterns, which should be considered during surgery. These findings will benefit our understanding of pelvic anatomy and enhance safety in performing radical surgery for treating pelvic diseases.

## Data Availability

The datasets used and/or analyzed during the current study are available from the corresponding author on reasonable request.

## Abbreviations

CIA: Common iliac artery
CIV: Common iliac vein
CT: Computed tomography
EIA: External iliac artery
EIV: External iliac vein
IGA: Inferior gluteal artery
IIA: Internal iliac artery
IIV: Internal iliac vein
IPA: Internal pudendal artery
IVC: Inferior vena cava
L5: 5th lumbar nerve
S1: 1st sacral nerve
S2: 2nd sacral nerve
SGA: Superior gluteal artery
SGV: Superior gluteal vein
